# Galectins use N-glycans of FGFs to capture growth factors at the cell surface and fine-tune their signaling

**DOI:** 10.1186/s12964-023-01144-x

**Published:** 2023-05-25

**Authors:** Aleksandra Gedaj, Dominika Zukowska, Natalia Porebska, Marta Pozniak, Mateusz Krzyscik, Aleksandra Czyrek, Daniel Krowarsch, Malgorzata Zakrzewska, Jacek Otlewski, Lukasz Opalinski

**Affiliations:** 1 Faculty of Biotechnology, Department of Protein Engineering, University of Wroclaw, Joliot-Curie 14a, 50-383 Wroclaw, Poland; 2 Faculty of Biotechnology, Department of Protein Biotechnology, University of Wroclaw, Joliot-Curie 14a, 50-383 Wroclaw, Poland

**Keywords:** FGF, Galectins, N-glycosylation, Multivalency, Signaling, Extracellular matrix, FGFR

## Abstract

Fibroblast growth factors (FGFs) and their receptors (FGFRs) constitute complex signaling hubs that are crucial for the development and homeostasis of the human body. Most of FGFs are released by cells using the conventional secretory pathway and are N-glycosylated, yet the role of FGFs glycosylation is largely unknown. Here, we identify N-glycans of FGFs as binding sites for a specific set of extracellular lectins, galectins − 1, -3, -7 and − 8. We demonstrate that galectins attract N-glycosylated FGF4 to the cell surface, forming a reservoir of the growth factor in the extracellular matrix. Furthermore, we show that distinct galectins differentially modulate FGF4 signaling and FGF4-dependent cellular processes. Using engineered variants of galectins with altered valency we demonstrate that multivalency of galectins is critical for the adjustment of FGF4 activity. Summarizing, our data reveal a novel regulatory module within FGF signaling, in which the glyco-code in FGFs provides previously unanticipated information differentially deciphered by multivalent galectins, affecting signal transduction and cell physiology.

Video Abstract

**Supplementary Information:**

The online version contains supplementary material available at 10.1186/s12964-023-01144-x.

## Background

**Fig. 1:**
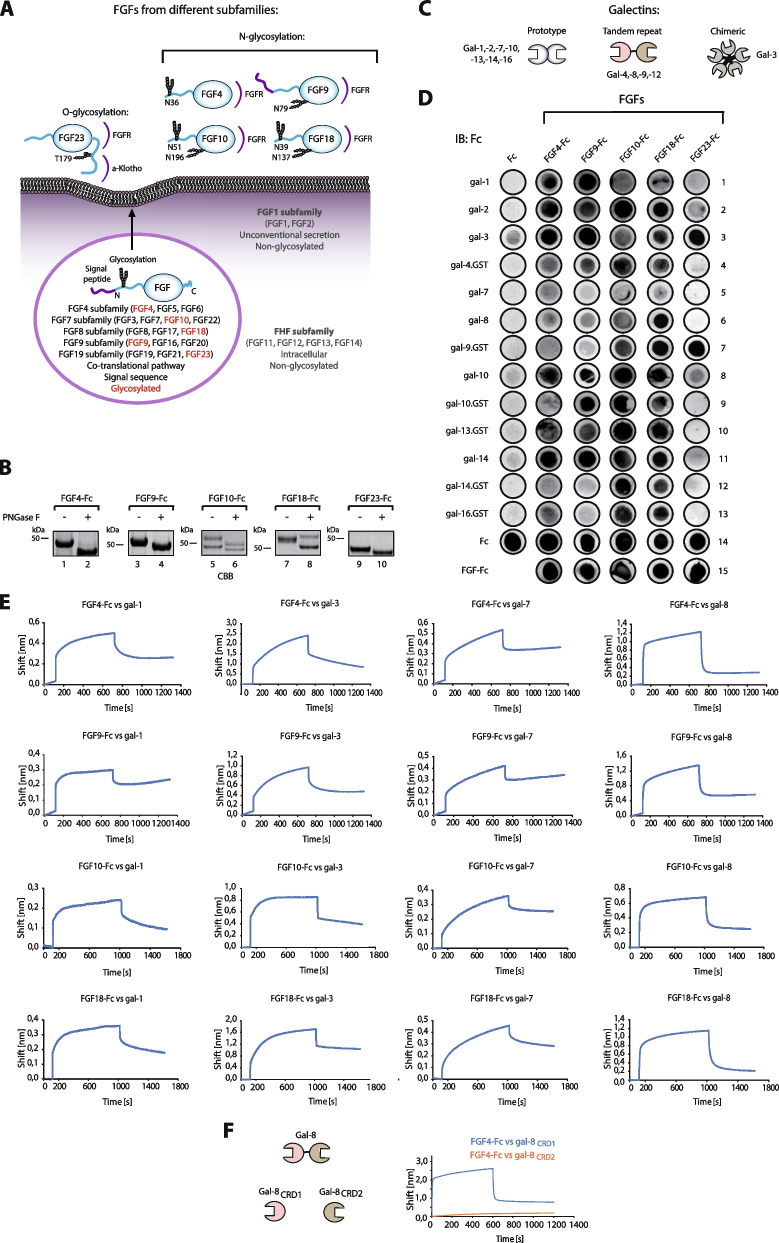
Galectins directly bind glycosylated FGFs.** A** Scheme of N-glycosylation among members of different FGF subfamilies. The FHF subfamily includes intracellular proteins that lack a secretion signal and thus do not undergo N-glycosylation. The FGF1 subfamily includes FGF1 and FGF2, which also lack the signal sequence for ER targeting, but are efficiently secreted *via* unconventional mechanisms bypassing ER/Golgi; these proteins are also devoid of N-glycosylation. The vast majority of FGFs (FGF4, FGF7, FGF8, FGF9, FGF19 subfamilies members) contain an N-terminal signal sequence for canonical secretion and many members of these families are N-glycosylated. In contrast. FGF23 is O-glycosylated and this modification controls stability of the growth factor. Representative members of all canonically secreted FGF subfamilies selected for experimental studies are marker in red. The predicted N- and O- glycosylation positions of the selected FGF members are shown. FGFR and KLA recognition regions are marked in purple. The propeller domain, typical of FGF proteins, is marker in blue. **B** CBB stained gels of PNGase F treated, representative N-glycosylated FGFs-Fc. **C** Schematic representation of the multivalent structures and classification of human galectins. Prototype galectins (galectin-1, -2, -7, -10, -13, -14, -16) contain a single carbohydrate recognition domain (CRD) that dimerizes. Tandem repeat galectins (galectin-4, -8, -9, -12) contain two distinct CRDs on a single polypeptide chain, while chimeric galectin-3 forms pentamers. **D** Galectin arrays to identify direct interactions between glycosylated FGFs and human galectins. Recombinant galectins were spotted onto the PVDF membrane and incubated with equimolar concentrations of the Fc fragment (control) and FGFs-Fc. After extensive washing, Fc-bearing proteins interacting with individual galectins were detected with anti-Fc-HRP antibodies and chemiluminescence. Representative results from at least three independent experiments are shown. **E** BLI analyses of the interaction between glycosylated FGFs and galectins. FGF4-Fc, FGF9-Fc, FGF10-Fc and FGF18-Fc were immobilized on Protein-A biosensors in a pairwise manner with equimolar concentrations of Fc and incubated with recombinant galectins to record the association and dissociation phases. Fc control values were subtracted from the signal obtained for FGFs-Fc. Representative results from at least three independent experiments are shown. **F** BLI measurements of the interaction of separate CRD1 and CRD2 of galectin-8 with FGF4-Fc

Fibroblast growth factors (FGFs) together with fibroblast growth factor receptors (FGFRs) transduce signals through the plasma membrane, regulating vital cellular processes, such as cell proliferation, differentiation, metabolism, motility and death [1]. FGF/FGFR activity is adjusted at multiple levels to support a large diversity of triggered cellular effects and to avoid pathological conditions that may lead to developmental diseases and cancers [2–4]. The human FGF family comprises 22 proteins divided into seven subfamilies [1]. Members of the FGF1 subfamily (FGF1 and FGF2) are produced without an N-terminal signal sequence and are secreted *via* unconventional mechanisms that bypass the ER/Golgi (Fig. 1A) [5–7]. The fibroblast growth factor homologous factors (FHF) (FGF11-14) group includes intracellular proteins and, so far, there is no evidence for their secretion [1]. In addition to these proteins, the vast majority of FGFs (FGF4, FGF7, FGF8, FGF9 and FGF19 subgroups) are produced with an N-terminal signal sequence that directs these proteins to the ER/Golgi prior secretion (Fig. 1A) [1]. Typically, these FGFs contain N-glycosylation motifs and, although the incorporation of N-linked sugar chains has been shown for several FGFs, the role of these modifications is largely unknown [8–17].

Galectins are small size, soluble lectins capable of multivalent binding of β-galactose containing glycoconjugates [18]. Galectins are found in the extracellular space and inside cells, and these proteins are involved in a wide range of cellular processes, such as signaling, endocytosis, autophagy, cell division, motility or apoptosis [19–21]. Human galectin family includes twelve members, which are divided into three groups based on their molecular architecture: prototypic (galectin-1,-2,-7,-10,-13,-14,-16), tandem-repeat (galectin-4,-8,-9,-12) and chimeric (galectin-3) (Fig. 1C) [22]. Prototypic galectins are composed of a single carbohydrate recognition domain (CRD) and are forming dimers. Tandem-repeat galectins comprise two different CRDs on a single polypeptide chain, while chimeric galectin-3, besides CRD, contains an N-terminal extension that facilitates high-order oligomerization (Fig. 1C) [18].

So far, the connection between galectins and FGF/FGFR signaling units has been limited to the plasma membrane-embedded components of FGF/FGFR signaling units: FGFRs and FGF/FGFR co-receptor: Klotho-β [19]. The association between FGF/FGFR and galectins was first reported by Ming et al., who demonstrated that galectin-3 moderates the accessibility of the Klotho-β co-receptor for the FGF21/FGFR1 signaling complex [23]. Galectin-3 also binds N-glycosylated integrin αvβ3 and modulates angiogenic activity of FGF2 [24]. N-glycosylation of FGFR1 is used by galectin-1 and − 3 to directly trigger FGFR1 signaling and alter cellular trafficking of the receptor [25, 26]. The role of extracellular galectins in the binding and modulation of N-glycans of secreted FGFs was so far fully unknown.

Here, we have performed the first in depth analysis of the interplay between all human galectins and representative members of canonically secreted N-glycosylated FGFs. We uncover the presence of a novel regulatory module in FGF/FGFR signaling, where N-glycosylation of FGFs constitutes a glyco-code that is differentially decoded by different members of the galectin family to fine-tune FGF/FGFR signaling and determine cell performance.

## Methods

### Antibodies and reagents

The primary antibodies directed against phospho-FGFR (pFGFR; #3476) and phospho-ERK1/2 (pERK1/2; #9101) were from Cell Signaling (Danvers, MA, USA). The anti-tubulin primary antibody (#T6557) was from Sigma-Aldrich (St. Louis, MO, USA). Anti-human IgG (Fc) antibody coupled to HRP (#ab97225) were from Abcam. Secondary antibodies were obtained from Jackson Immuno-Research Laboratories (Cambridge, UK). Protein A Sepharose, Glutathione Sepharose and Heparin Sepharose resins were from GE Healthcare (Chicago, IL, USA). Ni-NTA agarose and PrestoBlue™ Cell Viability Reagent (Thermo Fisher Scientific, Waltham, MA, USA).

### Cells

Mouse embryo fibroblast cells (NIH3T3) were cultured in Dulbecco’s Modified Eagle’s Medium - DMEM (Thermo Fisher Scientific,) supplemented with 10% fetal bovine serum (FBS) (Thermo Fisher Scientific) and antibiotics (100 U/mL penicillin, 100 µg/mL streptomycin). 3T3-L1 preadipocytes were maintained until 90% confluence. Next, the medium was exchanged to differentiation medium – DMEM (PAN-Biotech GmbH, Aidenbach, DE) supplemented with 10% FBS (Thermo Fisher Scientific), 0.5 mM isobutylmethylxanthine - IBMX (Sigma-Aldrich), 1 µg/mL insulin and 1 µM dexamethasone (Sigma-Al- drich), for 3 days. Next, adipocytes were maintained for maturation until day 12 in DMEM supplemented with 10% FBS and 1 µg/mL insulin. All cell lines were cultured in 5% CO2 atmosphere at 37 °C and were seeded onto tissue culture plates one day prior to the start of the experiments.

### Recombinant proteins

Genetic constructs allowing for expression of human galectins were prepared using the Gateway Cloning technique (according to the manufacturer’s protocol; Thermo Fisher Scientific). Galectins were expressed in *E. coli* BL21 CodonPlus(DE3)-RIL (Agilent Technologies, Santa Clara, CA, USA) at 16 °C (His-tagged galectins) or 25 °C (GST-tag galectins) overnight (details about plasmids and bacterial hosts used for galectin expression can be found in the Table S1). Galectins were isolated with the use of appropriate affinity chromatography. The proper folding of galectins was assessed by their ability to bind lactose agarose beads. His-tagged galectins were stored in PBS with 1 mM DTT, while GST-tagged galectins were stored in 50 mM Tris, 150 mM NaCl, 20 mM GSH, pH 7.5.

Plasmids for production of galectin truncations: Gal1_CRD_ (residues Ser^8^ – Asp^135^ of the wild type galectin-1), gal-8_CRD1_ (Met^1^ – Ser^152^), gal-8_CRD2_ (Phe^187^ – Trp^317^), gal-3_CRD_ (Gly^108^ – Leu^251^) were obtained using site directed mutagenesis with vectors enabling production of wild type proteins [26]. Construct for production of gal-1_CRD_.CC.5x was prepared with Restriction Free Cloning by insertion of coiled coil motif CC.5x to the gal-1_CRD_ expression vector [27, 28]. Engineered galectins were expressed in *E. coli* BL21(DE3) pLysS strain (Agilent Technologies, Santa Clara, CA, USA) and purified with affinity chromatography.

Genetic constructs allowing for the expression of FGF4-Fc, FGF9-Fc, FGF10-Fc, FGF18-Fc and FGF23-Fc were prepared in the mammalian expression vector pLEV113 using Restriction Free Cloning. All Fc fused FGFs were expressed in CHO cells and purified using Protein A Sepharose as described in [29]. FGF4-Fc protein was further purified by affinity chromatography using a HiTrap Heparin HP column (GE Healthcare). Elution of protein was performed with a NaCl gradient (in 25 mM HEPES pH 7.4, 1 mM DTT, 0.1 mM PMSF and 1 mM EDTA) in a range from 0 to 2 M using NGC Chromatography System (Bio-Rad, Hercules, CA, USA). FGF1 and FGF2 were purified as reported in [27, 30].

The purity and the identity of all obtained proteins were confirmed by SDS-PAGE and western blotting. The oligomeric state of recombinant proteins was assessed with gel filtration [27, 31].

### Galectin array with dot-blot

Recombinant galectins (0,5 pmol), FGFs and Fc (0,2 pmol), were dot-blotted onto a PVDF membrane, membranes were blocked with 3% BSA and galectins arrays were incubated with FGFs-Fc or the Fc (0,9 pM) overnight. Galectin-FGFR-Fc complexes were detected with anti-Fc mAb-HRP and chemiluminescence.

### Bio-Layer Interferometry (BLI)

To analyze the interaction between FGFs and galectins, BLI measurements were conducted using Octet RED K2 system (ForteBio, SanJose, CA, USA). FGFs-Fc and the Fc (25 µg/mL) were immobilized on Protein-A sensors in a pairwise manner (studied protein and the Fc on the reference sensor) and sensors were subsequently incubated with studied galectins (50 µg/mL). Signal for control (the Fc) was subsequently subtracted from the signal of FGF4-Fc. For measurements of binding kinetics sensor-immobilized FGF4-Fc (25 µg/mL) was incubated with various concentrations of galectins (0.2 µM to 6 µM). The heterogeneous ligand (2:1) model was used for data fitting using Data Analysis 11 Software (Fortebio). To evaluate the significance of FGF4-Fc N-glycosylation on its interaction with galectins, PNGase F-treated and non-treated FGF4-Fc (25 µg/mL) were immobilized on Protein-A biosensors and incubated with studied galectins (50 µg/mL).

### FGFR activation and downstream signaling cascades

Serum starved NIH3T3 cells were stimulated for 15 min with FGF1 (100 ng/mL) in the presence of heparin (10 U/mL) or various concentrations of recombinant galectins (1–5 µg/mL) and FGF4-Fc (2–20 ng/mL) or mixtures of studied proteins, at 37 °C. Cells were lysed in SDS-PAGE sample buffer, subjected to SDS-PAGE and visualized with western blotting using chemiluminescent substrate and ChemiDoc station (Bio-Rad). To study the impact of endogenous galectins on FGF4-Fc signaling, NIH3T3 cells were washed with 50 mM lactose in DMEM for 15 min directly before incubation with FGF4-Fc. Densitometric analysis of digital records was performed using the program ImageLab Software. At least three independent experiments were quantified.

### Cell proliferation and apoptosis

NIH3T3 were cultured in serum-free medium (DMEM) for 24 h. Cells were subsequently treated with galectin-1, -3, -7, -8 (5 µg/mL), FGF4-Fc (5 ng/mL) or mix of studied proteins. Then, cells were incubated at 37 °C, 5% CO2 for 48 h and cell proliferation was determined with Presto Blue Cell Viability Reagent (Thermo Fisher Scientific). At least three independent experiments were quantified. The effect of galectins on cell apoptosis was assessed with ApoLive-Glo Multiplex Assay (Promega), as described in [32].

### Glucose uptake

Differentiated 3T3-L1 cells seeded on the BioCoatTM Poly-D-Lysine 96-well (10,000 cells/well) (Corning, NY, USA) in DMEM without glucose (Thermo Fisher Scientific) and serum were stimulated with galectins (50 µg/mL), FGF4-Fc (108 ng/mL) or mixtures of these proteins for 16 h at 37 °C. The glucose uptake was determined with the Glucose Uptake-GloTM Assay (Promega, Madison, USA) according to the manufacturer’s protocol. At least three independent experiments were quantified.

### Fluorescence microscopy

Serum-starved U2OS-R1 cells were incubated in DMEM supplemented with heparin (40U/mL) with FGF4-Fc (20 µg/mL) in the presence or absence of galectins (20 µg/mL) for 30 min on ice to assess cell binding and at 37 °C for 30 min to study FGF4-Fc endocytosis. Cells were subsequently washed with ice cold PBS, fixed in 4% paraformaldehyde solution and permeabilized with 0.1% Triton in PBS (for cells incubated at 37 °C). Nuclei were stained with NucBlue Live dye (Thermo Fisher Scientific), Zenon AF-488 (Thermo Fisher Scientific) was used for detection of FGF4-Fc. Early endosomes were detected with rabbit anti-human polyclonal early endosome antigen 1 (EEA1) antibody (#ab2900, Abcam) and anti-rabbit IgG secondary antibody conjugated to Alexa Fluor 594 (#A11037, Thermo Fisher Scientific). Wide-field fluorescence microscopy was carried out using a Zeiss Axio Observer Z1 fluorescence microscope (Zeiss, Oberkochen, Germany) as described in [33]. Images were processed with Zeiss ZEN 2.3 software (Zeiss, Oberkochen, Germany), and Adobe Photoshop CS6 (Adobe, San Jose, CA, USA). For quantification of the cell binding by FGF4-Fc in the presence of galectin variants, the total fluorescence of at least 20 cells from three fields of view/condition was measured in three independent experiments using Zeiss ZEN 2.3 software.

### Statistics

Each of the experiments presented in the manuscript was repeated at least three times. Statistical analyses were performed with Student’s t-test (*p < 0.05; **p < 0.005 and ***p < 0.001).

## Results

### A specific set of galectins directly interact with FGFs

To study the interplay between secreted FGFs and galectins we produced representative members from all five FGF subgroups that are secreted in a conventional manner and contain N-glycosylation motifs as fusions with Fc in CHO cells: FGF4-Fc, FGF9-Fc, FGF10-Fc, FGF18-Fc and FGF23-Fc (Fig. 1B, lanes 1, 3, 5, 7 and 9). These FGFs contain either one (FGF4 and FGF9) or two (FGF10 and FGF18) putative N-glycosylation sites, which are located within the N-terminal extensions or in the FGF β-trefoil domain, in an area that based on the structures of the FGF/FGFR complexes is likely to be distant from the FGFR binding sites (Fig. 1A). Unlike the aforementioned FGFs, FGF23 lacks an N-glycosylation motif, but is O-glycosylated in the linker region between the FGFR and α-Klotho binding sites, and this modification regulates FGF23 activity (Fig. 1A) [14]. Since the Fc fragment of IgG1 can be N-glycosylated, we used FGF23-Fc as a control and demonstrated that after PNGase F treatment there is only a very slight change in the migration of FGF23-Fc in SDS-PAGE (which reflects de-glycosylation of the Fc) (Fig. 1B, lane 9 and 10). In contrast, PNGase F treatment largely altered the migration of FGF4-Fc, FGF9-Fc, FGF10-Fc and FGF18-Fc, confirming the N-glycosylation of these proteins (Fig. 1B, lanes 1–8).

The human galectin family consists of twelve proteins that differ in their oligomeric state and carbohydrate specificity (Fig. 1C). To screen for galectins that bind FGFs, we developed galectin dot blot arrays with recombinant human galectins [34]. FGFs-Fc, Fc (positive controls), and recombinant galectins were immobilized on PVDF membrane, membranes were blocked and the galectin arrays were incubated with equimolar concentrations of FGFs-Fc or Fc as a negative control. After extensive washing, complexes between individual galectin and FGFs were detected with anti-Fc antibody conjugated to HRP and chemiluminescence. The spots with positive controls: FGFs-Fc and Fc were readily detected with anti-Fc antibody, which confirms the validity of our experimental approach (Fig. 1D, rows 14 and 15). We observed positive signals for the vast majority of distinct galectins and FGF4-Fc, FGF9-Fc, FGF10-FC and FGF18-Fc (Fig. 1D). In contrast, for O-glycosylated FGF23-Fc we detected significantly fewer interactions with galectins, with positive signals identified mainly for galectin-3 and − 9 (Fig. 1D, rows 3 and 7). The presence of lactose in dot blot experiments with galectins greatly reduced the signal detected for most of galectins, while for few of galectins (mainly galectin-4, -13, -14 and − 16) the signal was enhanced, indicating their possible sugar-independent interaction with FGF4-Fc (Fig. S1). These data indicate that different galectins can directly interact with glycosylated FGFs.

To confirm these findings, we used biolayer interferometry (BLI). FGFs-Fc and the Fc fragment were immobilized on Protein-A BLI biosensors in a pairwise manner (FGF-Fc and Fc as a control), incubated with recombinant galectins and the association and dissociation stages of the interaction between the proteins tested were monitored. For FGF4-Fc, FGF9-Fc, FGF10-Fc and FGF18-Fc, the strongest signals were obtained for galectin-1, -3, -7 and − 8 (Fig. 1E). For O-glycosylated FGF23-Fc, we detected virtually no interaction with galectin-1, -3 and − 7 and a weak signal for galectin-8 and − 13 (Fig. S2). The presence of lactose completely inhibited the binding of galectin-1, -3, -7 and − 8 to FGF4-Fc (Fig. S3). We also confirmed with BLI that the interaction with GST-tagged FGF4-Fc was not due to non-specific binding to GST (Fig. S4).

Galectin-8 is a tandem repeat galectin composed of two CRDs with distinct carbohydrate specificities (Fig. 1C) [35]. We produced separate CRDs of galectin-8 and demonstrated that only gal-8_CRD1_ interacts with FGF4-Fc, indicating that CRD1 is exclusively involved in FGF4-Fc binding (Fig. 1F).

These data indicate that galectins from all three families directly interact with all tested N-glycosylated FGFs, while they display very limited binding to O-glycosylated FGF23. Since galectin-1, -3, -7 and − 8 showed positive signals in both galectin dot-blots and BLI, we decided to focus on these galectins in subsequent studies.

### N-glycans of FGFs constitute binding sites for galectins

**Fig. 2:**
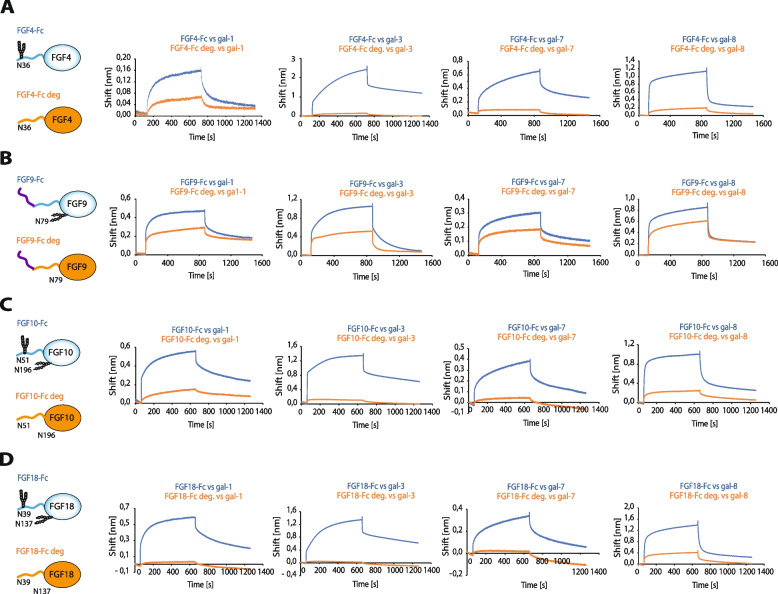
N-glycans of FGFs are recognized by galectins. **A-D** BLI analyses of the interaction between N-glycosylated and de-glycosylated FGFs and galectins. N-glycosylated and enzymatically de-glycosylated FGFs: FGF4-Fc (**A**), FGF9-Fc (**B**), FGF10-Fc (**C**) and FGF18-Fc (**D**) were immobilized on Protein-A biosensors (blue lines) in a pairwise fashion with equimolar concentrations of PNGase F-treated FGF4-Fc, FGF9-Fc, FGF10-Fc and FGF18-Fc, respectively (red lines), and the association and dissociation phases were recorded with BLI. Representative results from at least three independent experiments are shown

We evaluated whether the direct interaction between galectin-1, -3, -7 and − 8 and FGFs occurs *via* N-glycans of FGFs. BLI experiments with enzymatically de-glycosylated FGFs-Fc revealed that FGF4, FGF10 and FGF18 strictly require N-glycosylation for binding of galectins (Fig. 2A, C and D). However, we observed a weaker dependence on N-glycosylation for the interaction between FGF9-Fc and galectins (Fig. 2B). These data indicate that N-glycans of FGFs are recognized by galectin-1, -3, -7 and − 8.

Subsequently, for reasons of ease of isolation and handling, we decided to focus on FGF4-Fc as a representative N-glycosylated FGF and studied in depth its functional interconnection with identified galectins. We measured kinetic parameters of the interaction between FGF4-Fc and galectins. As shown in Table 1 galectins bind FGF4 with affinity in high nanomolar range (K_D_ of about 10^− 7^ M).

**Table 1:** Kinetic parameters of the interaction between the studied galectins and FGF4

	K_D1_ [M]	K_D2_ [M]	K_on1_[M^− 1^ s^− 1^]	K_on2_[M^− 1^ s^− 1^]	K_Off1_ [s^− 1^]	K_Off2_ [s^− 1^]
**gal-1**	9.1E-07^a^					
**gal-3**	1.42E-07	1.07E-06	1.39E + 03	5.02E + 05	1.97E-04	5.38E-01
**gal-7**	2.68E-07	4.97 E-07	1.21 E + 03	1.20E + 05	3.23E-04	5.96E-02
**gal-8**	7.24E-07	1.40E-07	1.04E + 05	8.23E + 02	7.55E-02	1.15E-04

^a^for the interaction of galectin-1 with FGF4, as experimental data did not fit the kinetic models, steady-state analysis was used to determine Kd. Two values of K_D_, k_on_ and k_off_ were obtained from a 2:1 heterogenous ligand model, which was used to fit experimental kinetics data fitting

### Multivalency of galectins is strictly required for differential fine-tuning of the trafficking and signaling of N-glycosylated FGF4

**Fig. 3:**
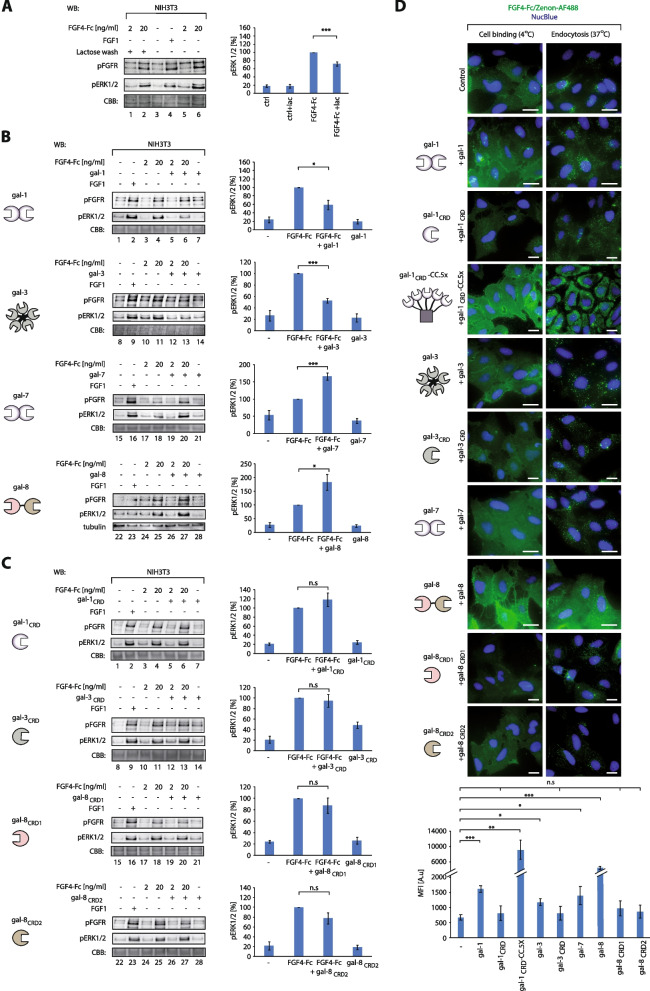
Multivalency of galectins is crucial for the attraction of glycosylated FGF4-Fc to the cell surface and for the modulation of FGF4-Fc trafficking and signaling.** A** Effect of endogenous galectins on FGF4-Fc signaling. Serum-starved NIH3T3 cells were washed with 50 mM lactose prior cell supplementation with various concentrations of FGF4-Fc (2–20 ng/mL). Cells were lysed and analyzed with WB using the indicated antibodies. CBB served as a loading control (left panel). Densitometric analyses of the effect of lactose washes on the activation of FGF-Fc-dependent signaling pathways by 20 ng/mL FGF4-Fc (right panel). Mean values from at least three independent experiments +/- SEM are shown. Statistical analyses were performed with Student’s t-test (*p < 0.05; **p < 0.005 and ***p < 0.001). **B **and** C** Effects of the wild type galectins (**B**) and their monovalent variants (**C**) on FGF4-Fc signaling. Serum-starved NIH3T3 cells were treated with FGF1 (100 ng/mL, control), different concentrations of FGF4-Fc (2 and 20 ng/mL) in the presence or absence of recombinant galectins (1 µg/mL) or their monovalent variants (5 µg/mL). Cells were lysed and analyzed with WB using the indicated antibodies (left panel). CBB and tubulin served as loading controls. Densitometric analyses of the effect of galectins on FGF4-Fc (20 ng/mL) signaling (right panel). Mean values from at least three independent experiments +/- SEM are shown. Statistical analyses were performed with Student’s t-test (*p < 0.05; **p < 0.005 and ***p < 0.001). The schemes of the wild type galectins and their engineered variants with altered valency are shown (left panel). **D** Effects of the wild type galectins and their engineered variants with altered valency on the cell binding and endocytosis of FGF4-Fc. U2OS-R1 cells were incubated for 30 min with FGF4-Fc (20 µg/mL) either at 4 °C (for cell binding analysis) or at 37 °C (for growth factor endocytosis analysis) in the presence of the studied galectins and their variants (20 µg/mL). Cells were either fixed (4 °C samples) or fixed and permeabilized (37 °C samples), nuclei were labelled with NucBlue and FGF4-Fc was detected with Zenon-AF-488 using fluorescence microscopy. Representative images from at least three independent experiments are shown. Scale bars represent 20 μm. To quantify cell binding by FGF4-Fc in the presence of galectin variants, the total fluorescence of at least 20 cells from three fields of view/condition was measured in three independent experiments using Zeiss ZEN 2.3 software. Statistical analyses were performed with Student’s t-test (*p < 0.05; **p < 0.005 and ***p < 0.001). The schemes of the wild type galectins and their engineered variants with altered valency are shown (left panel)

Since we identified a direct interaction of galectin-1, -3, -7 and − 8 with the N-linked sugars of FGFs, we wondered whether galectins could modulate the trafficking, signaling and function of N-glycosylated FGF4. To investigate whether endogenous galectins adjust the signaling of FGF4-Fc, we washed serum-starved NIH3T3 cells with lactose and treated the cells with FGF4-Fc. Lactose washes significantly reduced FGF4-Fc ability to induce phosphorylation of FGFR1 and ERK1/2, indicating that endogenous extracellular galectins modulate FGF4 (Fig. 3A). We assessed the effect of particular identified galectins on the ability of FGF4-Fc to activate FGFR-dependent signaling by pre-forming FGF4-Fc complexes with the galectins tested, incubating the complexes with cells and evaluating pFGFR and pERK1/2 levels. Interestingly, galectin-1 and − 3 significantly inhibited the activation of FGFR1 and ERK1/2 by FGF4-Fc, while galectin-7 and − 8 increased receptor activation and upregulated downstream signaling cascades (Fig. 3B).

To evaluate whether multivalency of galectins is required for modulation of FGF4-Fc signaling we produced truncated variants of galectin-1, -3 including solely CRD domains and lacking regions responsible for oligomerization [18, 36, 37]. CRD domains of galectin-1 and − 3, in contrast to the wild type proteins, had no effect on FGF4-Fc signaling (Fig. 3C). Similarly, uncoupled CRD1 and CRD2 domains of galectin-8 displayed no FGF4-Fc stimulatory activity (Fig. 3C).

Using fluorescence microscopy, we evaluated the effect of wild type galectins and their monovalent variants on the efficiency of FGF4-Fc binding and internalization by cells. We observed increased FGF4-Fc interaction with cells in the presence of all tested galectins (Fig. 3D). The strongest increase in the signal of FGF4-Fc at the cell surface was found when the growth factor was co-incubated with galectin-8 (Fig. 3D). Importantly, the increased binding of FGF4-Fc to cells strictly required the multivalency of galectins, as their monomeric engineered variants: gal-1_CRD_, gal-3_CRD_, gal-8_CRD1_ and gal-8_CRD2_ were unable to enhance FGF4-Fc interaction with cells (Fig. 3D). To further prove that multivalency of galectins is required for their action on FGF4-Fc, we engineered pentavalent galectin-1, gal-1_CRD_-CC.5x, by fusing gal-1_CRD_ with coiled coil motif enabling pentamerization [27]. We detected the massive accumulation of FGF4-Fc at the cell surface after co-incubation with multivalent gal-1_CRD_-CC.5x (Fig. 3D).

When FGF4-Fc was incubated with cells at 37 °C, the growth factor was effectively endocytosed and no cell surface-bound FGF4-Fc signal was detected, which we confirmed by co-localizing the intracellular FGF4-Fc signal with an early endosome marker protein, EEA1 (Fig. S5). FGF4-Fc internalization was differentially adjusted by the galectins tested, where galectin-1 and − 3 enhanced the efficiency of FGF4-Fc internalization, while galectin-7 had no significant effect on cellular uptake of FGF4-Fc (Fig. 3D). In the case of galectin-8, in addition to numerous spots indicating endosomes with internalized FGF4-Fc, we observed a strong signal of FGF4-Fc remaining on the cell surface (Fig. 3D). Internalization of FGF4-Fc was not affected by the monovalent engineered galectins: gal-8_CRD1_, gal-8_CRD2_, gal-1_CRD_ and gal-3_CRD_, while gal-1_CRD_-CC.5x, like the wild type galectin-8, caused, additionally to intracellular FGF4-Fc spots, partial accumulation of FGF4-Fc on the cell surface (Fig. 3D).

**Fig. 4:**
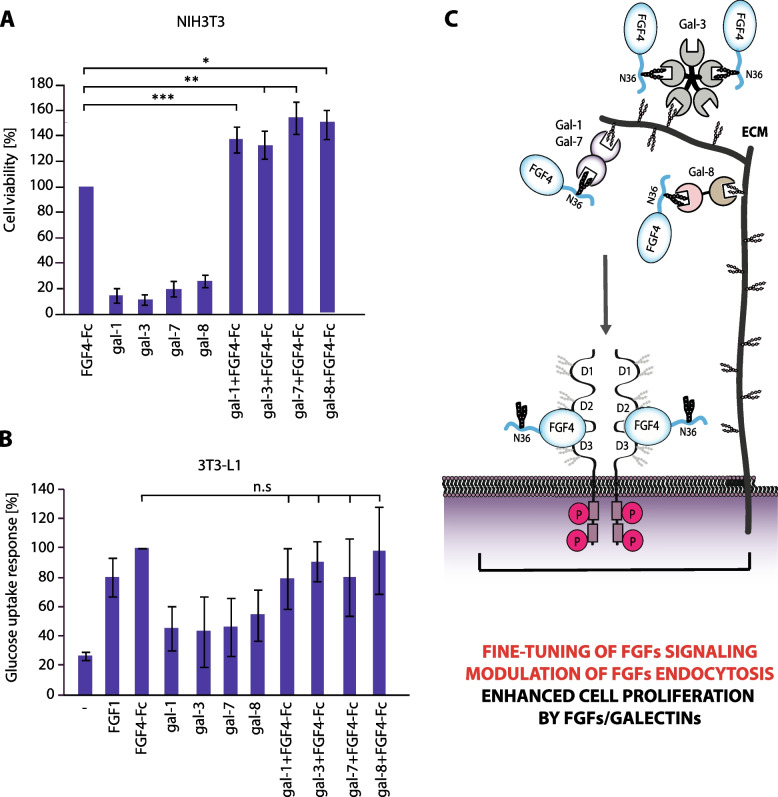
Galectins modulate cellular activities of FGF4.** A** The effect of FGF4-Fc (5 ng/mL), galectins (5 µg/mL) and a mixture of FGF4-Fc (5 ng/mL)/ galectin (5 µg/mL) on NIH3T3 cell proliferation assessed with the Presto Blue Cell Viability Reagent. Mean values +/-SD from at least three independent experiments are shown. Statistical analyses were performed with Student’s t-test (*p < 0.05; **p < 0.005 and ***p < 0.001). **B** Effects of FGF4-Fc (108 ng/mL), galectins (50 µg/mL) and a mixture of FGF4-Fc (108 ng/mL) / galectin (50 µg/mL) on glucose uptake by adipocytes. Mean values +/-SD from at least three independent experiments are shown. Statistical analyses were performed with Student’s t-test (*p < 0.05; **p < 0.005 and ***p < 0.001). **C** A hypothetical model of the modulation of FGF4 signaling by galectins. Simultaneous binding of multivalent galectins to the N-glycans of FGF4 and to yet unidentified extracellular matrix (ECM) components attracts FGF4 to the cell surface, thereby forming a reservoir of growth factor in the vicinity of plasma membrane-embedded FGFRs. Galectin-facilitated cell binding is reflected in altered FGF4/FGFR signaling and endocytosis, which ultimately shapes cell division without affecting metabolic activity of cells. The diversity of galectins’ effects on FGF4 is likely determined by different binding partners of particular galectins in the ECM

We next determined the cellular consequences of galectin-induced changes in FGF4 signaling. We demonstrated that FGF4-Fc signaling results in highly efficient stimulation of NIH3T3 cell division (Fig. 4A). The combination of the studied galectins with FGF4-Fc significantly enhanced the mitogenic activity of the cells, and this effect was completely abolished by the presence of lactose (Fig. 4A, Fig. S6). We also observed that galectins alone could also inhibit apoptosis in a dose-dependent manner (Fig. S7) and improve viability of cells even at high concentration (Fig. S8). Galectins have been previously implicated in regulation of cellular glucose uptake [38]. Although FGF4-Fc and galectins alone stimulated glucose uptake by 3T3 adipocytes, FGF4-Fc/galectin mixtures displayed the same metabolic activity as FGF4-Fc alone (Fig. 4B).

All these data indicate that galectins, through binding to FGF4 N-glycans, modulate cell binding, signaling and growth factor internalization, and adjust FGF4-dependent cellular processes. Moreover, our data suggest that the multivalency of galectins is critical for their activity towards N-glycosylated FGF4.

## Discussion

The vast majority of FGFs are N-glycosylated during their secretion and typically N-glycosylation sites of FGFs are located far from FGFR binding sites, either on unstructured N-terminal extensions or on FGF β-trefoil domain located opposite to FGFR-recognizing residues (Fig. 1A). Here, as first ones we identified a set of galectins that directly interact with sugar chains attached to model FGFs from all five FGF subfamilies secreted in the conventional manner. Galectins are highly abundant components of the extracellular matrix and their levels often correlate with cancer development and progression [21, 39, 40]. In this report, we have elucidated a novel mechanism for regulating FGF signaling, in which multivalent galectins utilize information stored in the N-glycans to capture FGFs in the extracellular matrix (Fig. 4C). Since multivalency of galectins is strictly required for galectins action on a single N-glycosylated FGF4, we hypothesize that one CRD of multivalent galectins is used to directly bind FGF4 N-glycans, while the other CRD recognizes other glycosylated cell surface components, presumably the extracellular matrix (ECM), thus serving as an anchor for the FGF4/galectin complex (Fig. 4C). Consistent with this, galectins are well-known interactors of several ECM proteins [41–51]. In this way, galectins may form a reservoir of growth factor in the vicinity of FGFRs, resembling the action of heparan sulphate proteoglycans (HSPGs), well described components of FGF/FGFR signaling units [52, 53]. Galectins differentially alter the amplitude of FGFR signals triggered by FGF4 and modify growth factor endocytosis. The distinctive effects of galectins on FGF4 signaling and trafficking likely depend on the properties of the ECM components to which FGF4 is clustered by a given galectin and kinetics of FGF4 release and re-location to FGFRs. Importantly, galectins shape FGF4-dependent cellular processes by promoting cell proliferation, while having no significant effect on cellular glucose uptake.

## Conclusions

Our data define a novel module in FGF signaling, where information stored in the N-glycans of FGFs is differentially read by many different galectins and translated to alter cell signaling and physiology. In agreement with our findings, Gordon-Alonso et al. recently reported that galectin-3 recognizes N-glycans of interferon-gamma and captures it in the tumor matrix, facilitating tumor evasion [54]. The formation of N-glycan-independent heterocomplexes between the chemokine CXCL12 and galectin-3 has been recently revealed and shown to modulate inflammatory processes [55, 56]. As ligands of other RTKs are also glycosylated, it is tempting to speculate that galectins may represent complex regulators of a wide range of growth factors and their receptors.

## Supplementary Information

**Additional file 1.**

## Data Availability

All data and materials are available from the corresponding author upon a reasonable request.

## Abbreviations

FGFRs: fibroblast growth factor receptors
FGFs: fibroblast growth factors
HSPGs: heparan sulphate proteoglycans
CRD: carbohydrate recognition domain
FHF: fibroblast growth factor homologous factor
BLI: biolayer interferometry
CC: coiled coil
DMEM: Dulbecco's Modified Eagle's Medium
FBS: fetal bovine serum
