# Correction: Galectins use N-glycans of FGFs to capture growth factors at the cell surface and fine-tune their signaling

**DOI:** 10.1186/s12964-023-01192-3

**Published:** 2023-06-13

**Authors:** Aleksandra Gedaj, Dominika Zukowska, Natalia Porebska, Marta Pozniak, Mateusz Krzyscik, Aleksandra Czyrek, Daniel Krowarsch, Malgorzata Zakrzewska, Jacek Otlewski, Lukasz Opalinski

**Affiliations:** 1 Faculty of Biotechnology, Department of Protein Engineering, University of Wroclaw, Joliot-Curie 14a, 50-383 Wroclaw, Poland; 2 Faculty of Biotechnology, Department of Protein Biotechnology, University of Wroclaw, Joliot-Curie 14a, 50-383 Wroclaw, Poland

**Correction: Cell Commun Signal 21, 122 (2023)**

**https://doi.org/10.1186/s12964-023-01144-x**

Following publication of the original article [1], the authors reported a typesetting error in the title of their article. The words “Short report” were erroneously included in the title. This correction article reflects the correct title and the original article [1] has been corrected.
