# Supplementary material for: Galectins use N-glycans of FGFs to capture growth factors at the cell surface and fine-tune their signaling
Source: Cell Commun Signal. 2023 May 25;21:122. doi: 10.1186/s12964-023-01144-x (PMC10214663; doi:10.1186/s12964-023-01144-x)
Supplement: Supplementary file 2 — Additional file 1. [file 12964_2023_1144_MOESM1_ESM.docx]

Supplementary Information for:

**Galectins use N-glycans of FGFs to capture growth factors at the cell surface and fine-tune their signaling**

Aleksandra Gedaj^1^, Dominika Zukowska^1^, Natalia Porebska^1^, Marta Pozniak^1^, Mateusz Krzyscik^1^, Aleksandra Czyrek^2^, Daniel Krowarsch^2^, Malgorzata Zakrzewska^1^, Jacek Otlewski^1^ and Lukasz Opalinski^1^*

^1^Faculty of Biotechnology, Department of Protein Engineering, University of Wroclaw, Joliot-Curie 14a, 50-383 Wroclaw, Poland

^2^Faculty of Biotechnology, Department of Protein Biotechnology, University of Wroclaw, Joliot-Curie 14a, 50-383 Wroclaw, Poland

*Correspondence should be addressed to L.O ([lukasz.opalinski@uwr.edu.pl](mailto:lukasz.opalinski@uwr.edu.pl))

**Supplementary Figure Legends:**

**Fig. S1. Galectin dot blot experiments with FGF4-Fc in the presence of lactose.** Recombinant galectins were spotted onto the PVDF membrane and incubated with FGF4-Fc in the presence or absence of 50 mM lactose. After extensive washing, FGF4-Fc interacting with individual galectins was detected with anti-Fc-HRP antibodies and chemiluminescence.

**Fig. S2. BLI measurements of the interaction between FGF23-Fc and galectin-1, -3, -7, -8 and -13.** FGF23-Fc was immobilized on Protein-A biosensors in a pairwise manner with equimolar concentrations of Fc and incubated with recombinant galectins to record the association and dissociation phases. Fc control values were subtracted from the signal obtained for FGFs-Fc.

**Fig. S3. BLI measurements of galectins’ interaction with FGF4-Fc in the presence of lactose.** FGF4-Fc (10 μg/mL) was immobilized on BLI biosensors and incubated recombinant galectin-1, -3, -7 and -8 in PBS (control) or in PBS with 25 mM lactose.

**Fig. S4. BLI measurements of the interaction between GST-galectin-9 and GST.** FGF4-Fc (10 μg/mL) was immobilized on BLI biosensors and incubated with recombinant GST-galectin-9 (10 μg/mL) or GST (10 μg/mL).

**Fig. S5. Fluorescence microscopy analysis of FGF4-Fc co-localization with EEA1.** Serum-starved U2OS-R1 cells were incubated in DMEM supplemented with heparin (40U/mL) and FGF4-Fc (20 μg/mL) for 30 min. Cells were subsequently washed with ice cold PBS, fixed in 4% paraformaldehyde solution and permeabilized with 0.1% Triton in PBS. Nuclei were stained with NucBlue Live dye, Zenon AF-488 was used for detection of FGF4-Fc. Early endosomes were detected with rabbit anti-human polyclonal antibody specific for early endosome antigen 1 (EEA1) and anti-rabbit IgG secondary antibody conjugated to Alexa Fluor 594. Scale bar represents 20 μm.

**Fig. S6. The effect of lactose on cell proliferation induced by FGF4-Fc/galectins.** The effect of 50 mM lactose on the NIH3T3 cell proliferation induced by a mixture of FGF4-Fc (5 ng/mL)/ galectin (5 μg/mL) assessed with the Presto Blue Cell Viability Reagent.

**Fig. S7. Antiapoptotic activity of galectins.** Ratio of caspase 3/7 activity in serum-starved NIH3T3 cells stimulated with distinct concentrations of galectin-1, -3, -7 and -8 to cell viability. The data shown are mean values ±SD of four independent experiments normalized toward untreated cells.

**Fig. S8. Cell viability upon treatment with high concentrations of galectins.** NIH3T3 cells were stimulated for 24h with galectins (20 μg/mL and 50 μg/mL) and cell viability was assessed with Presto Blue Cell Viability Reagent.

**
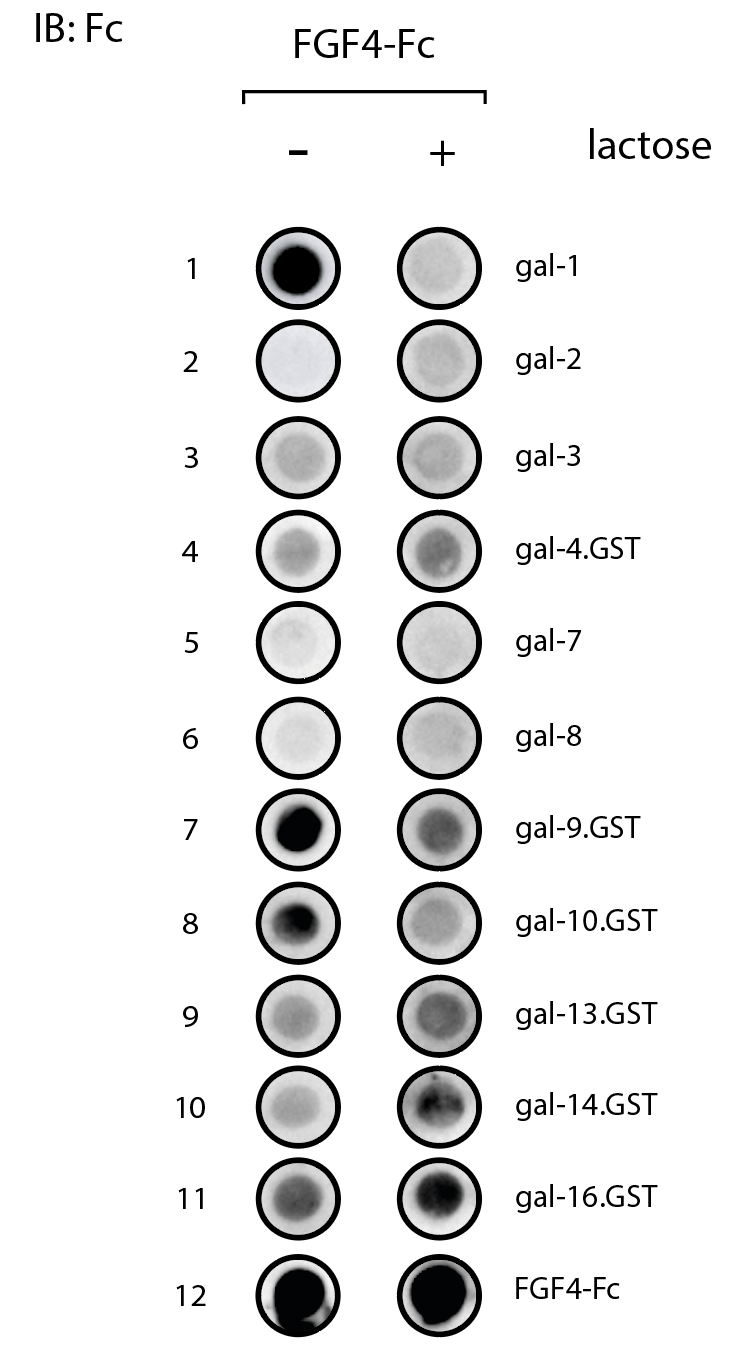
**

**Figure S1**

**
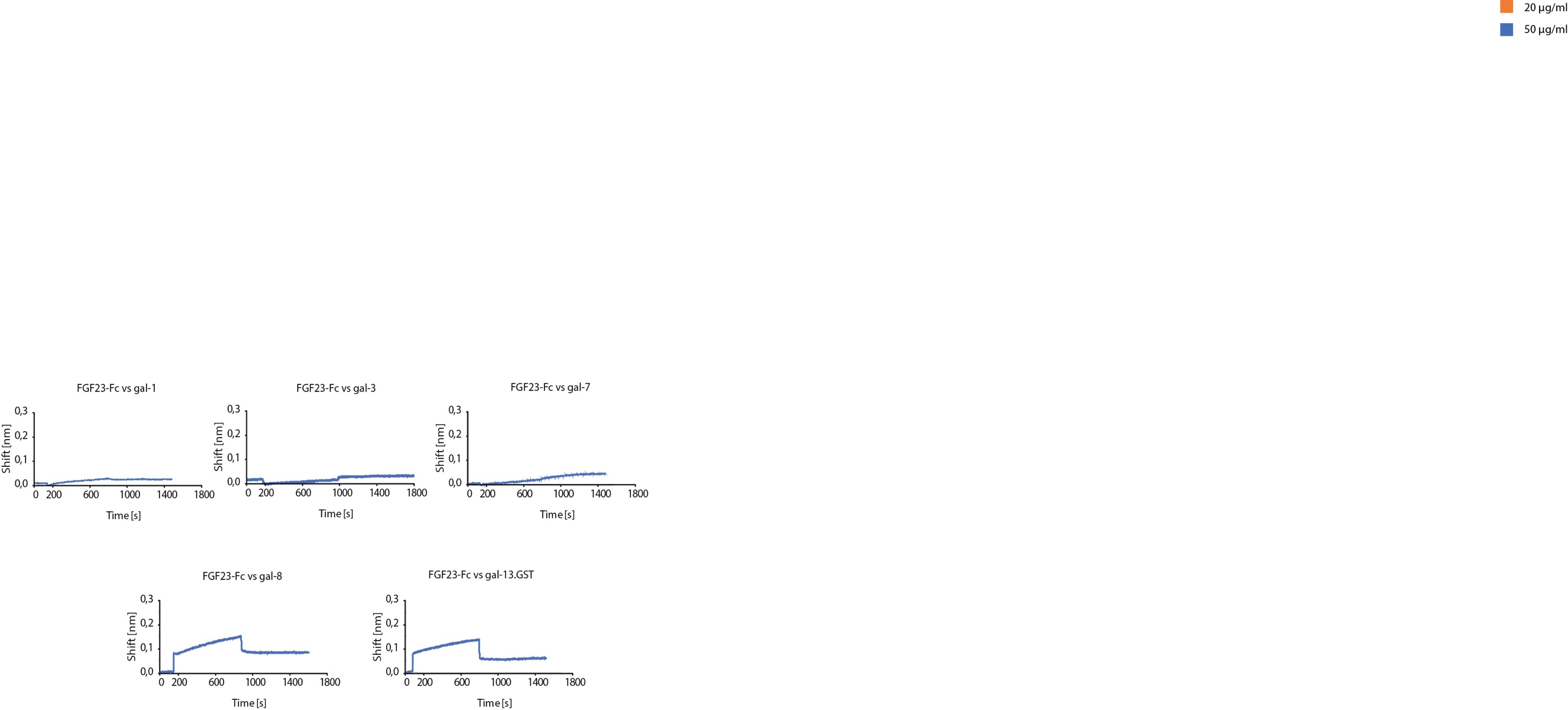
**

**Figure S2**

**
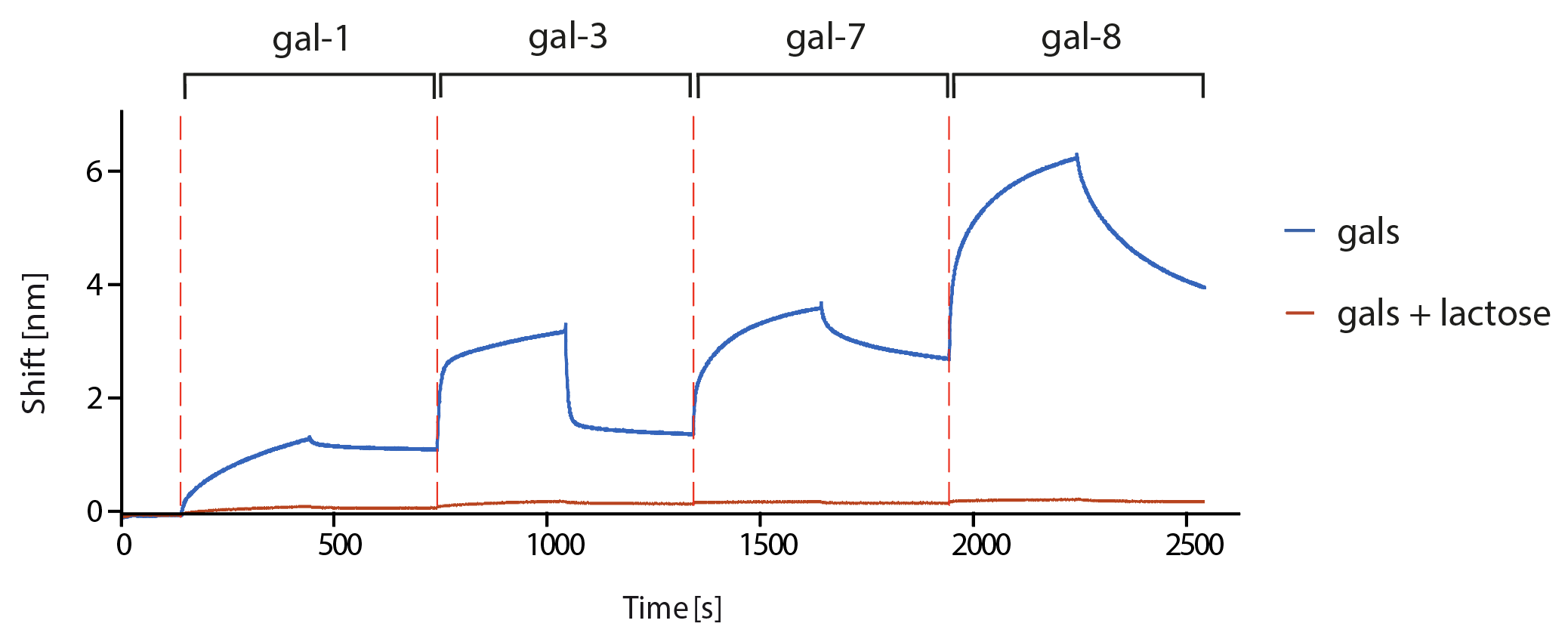
**

**Figure S3**

**
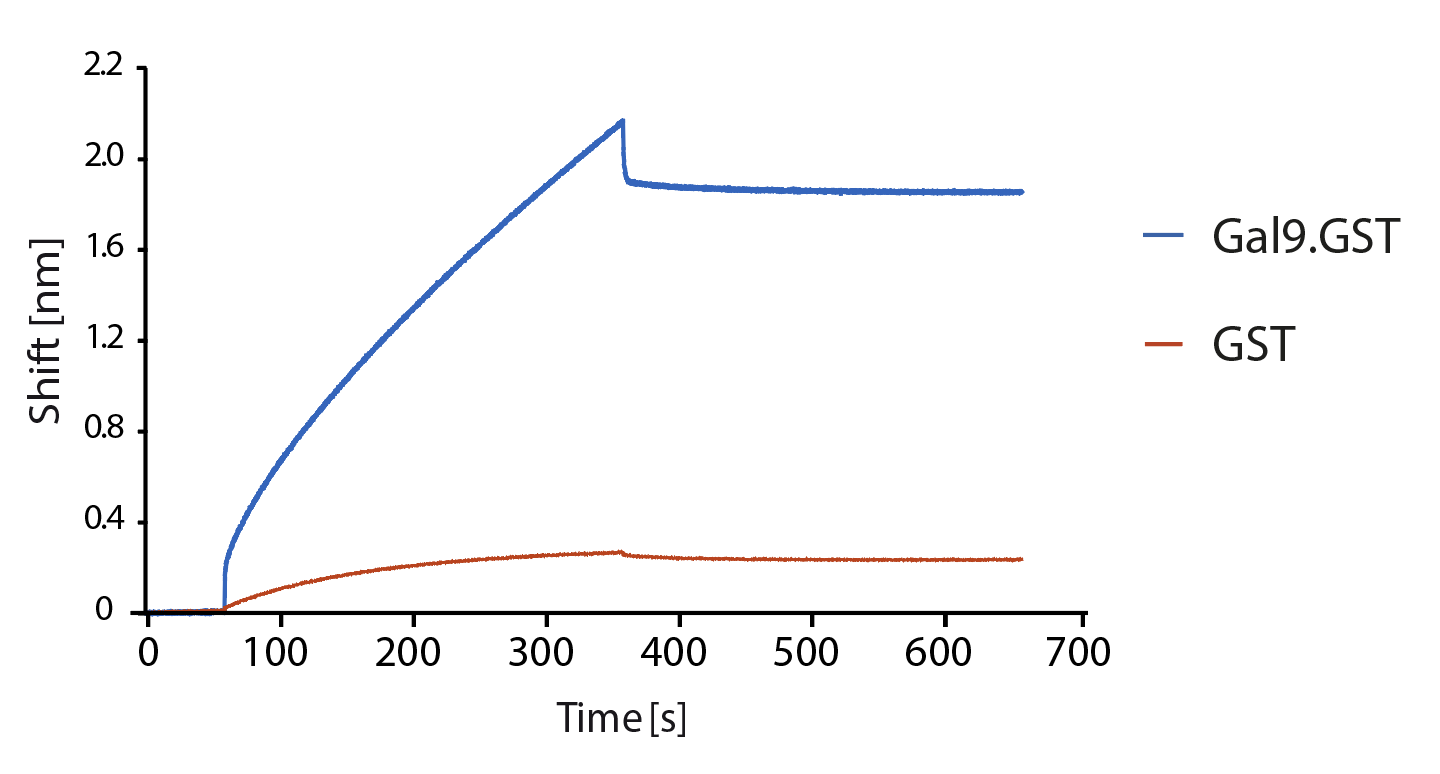
**

**Figure S4**

**
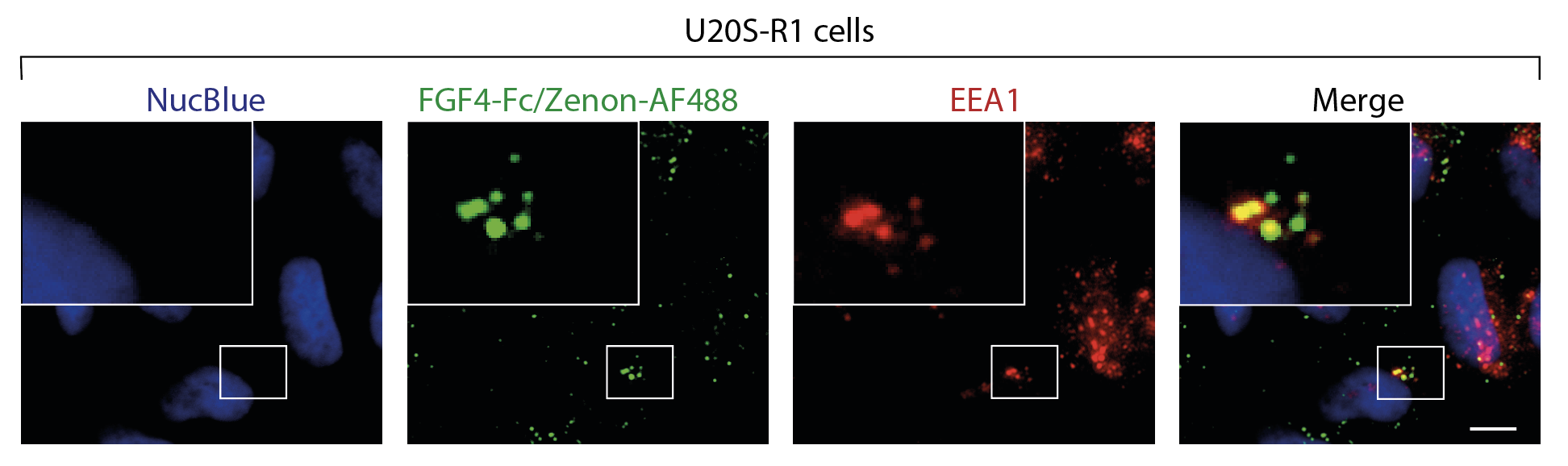
**

**Figure S5**


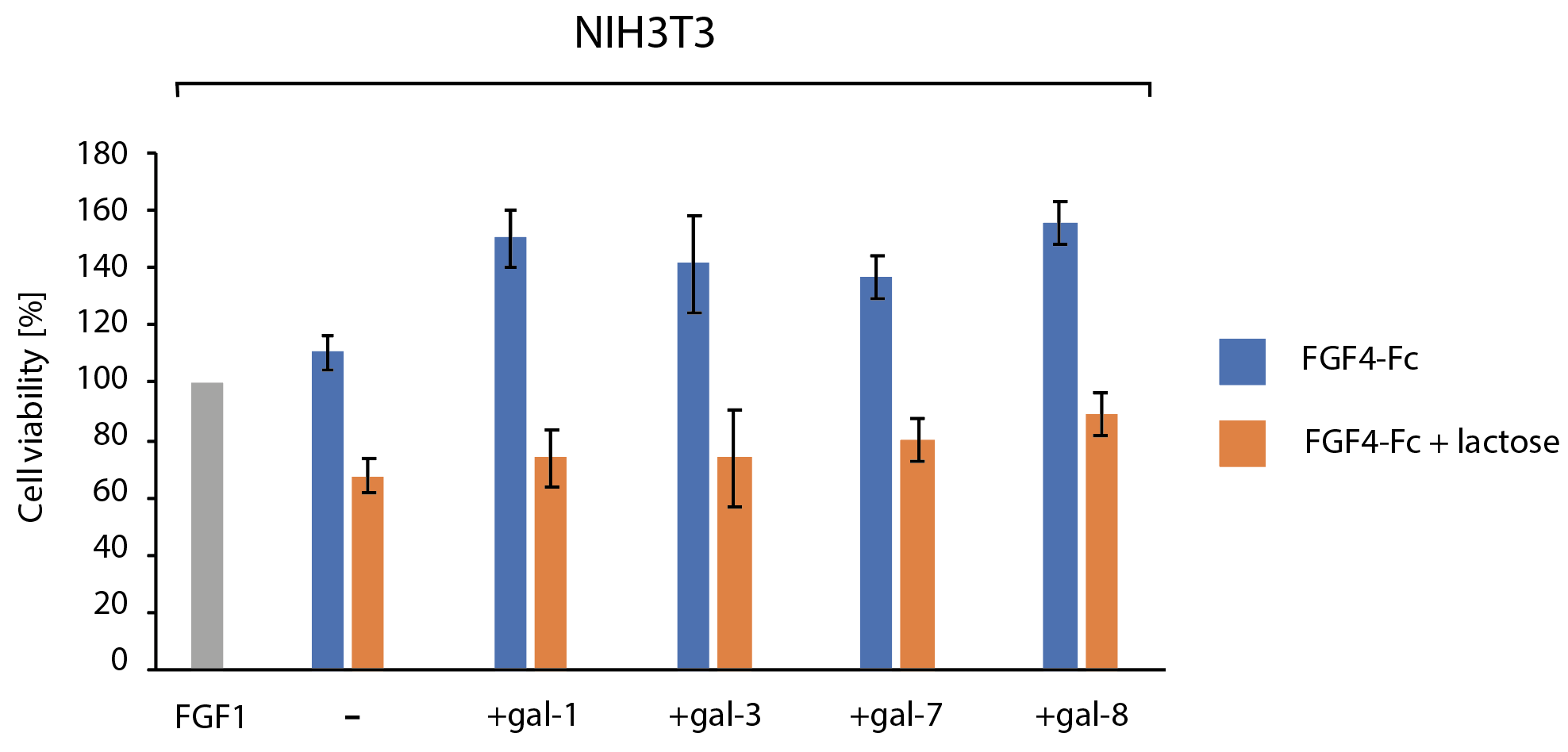


**Figure S6**

**
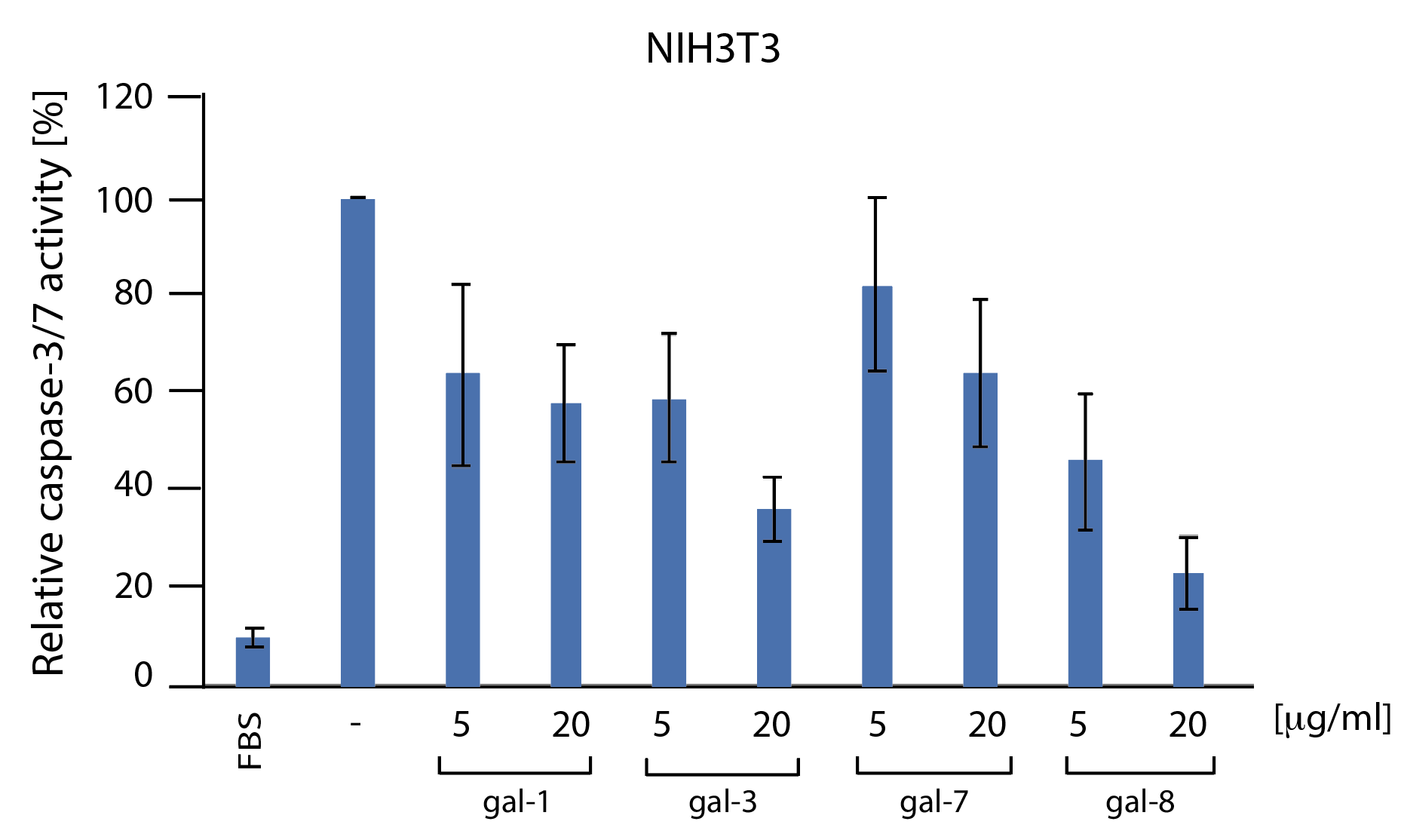
**

**Figure S7**

**
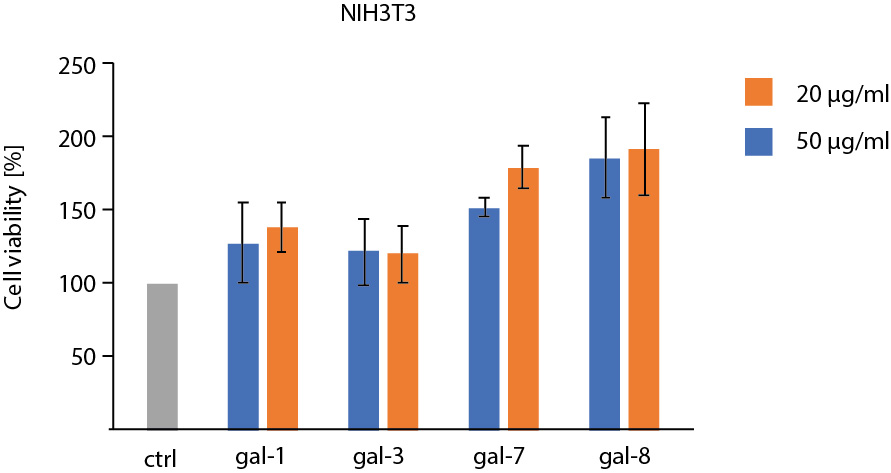
**

**Figure S8**

**Table S1. Plasmids, bacterial strains and expression conditions used for production of recombinant galectins.**

| **Protein name** | **Plasmid** | ***E. coli* strain** | **Expression conditions** |
| --- | --- | --- | --- |
| Galectin-1 | pETM11 | BL21 CodonPlus (DE3)-RIL | LB media, 37°C, 4h |
| Galectin-2 | pDEST17 | BL21 (DE3)-pLysS | LB media,16°C, ON |
| Galectin-2.GST | pDEST15 | BL21 (DE3)-pLysS | LB media,16°C, ON |
| Galectin-3 | pETM11 | BL21 CodonPlus (DE3)-RIL | LB media, 37°C, 4h |
| Galectin-4.GST | pDEST15 | BL21 CodonPlus (DE3)-RIL | LB media, 25°C, ON |
| Galectin-7 | pDEST17 | BL21 CodonPlus (DE3)-RIL | LB media,16°C, ON |
| Galectin-8 | pDEST17 | BL21 CodonPlus (DE3)-RIL | LB media,16°C, ON |
| Galectin-9.GST | pDEST15 | BL21 CodonPlus (DE3)-RIL | LB media, 25°C, ON |
| Galectin-10 | pDEST17 | BL21 CodonPlus (DE3)-RIL | LB media,16°C, ON |
| Galectin-10.GST | pDEST15 | BL21 CodonPlus (DE3)-RIL | LB media, 25°C, ON |
| Galectin-13.GST | pDEST15 | BL21 CodonPlus (DE3)-RIL | LB media, 25°C, ON |
| Galectin-14 | pDEST17 | BL21 (DE3)-pLysS | LB media,16°C, ON |
| Galectin-14.GST | pDEST15 | BL21 (DE3)-pLysS | LB media,16°C, ON |
| Galectin-16 | pDEST17 | BL21 CodonPlus (DE3)-RIL | LB media,16°C, ON |
| Galectin-16.GST | pDEST15 | BL21 CodonPlus (DE3)-RIL | LB media, 25°C, ON |
